# Prehospital Point-Of-Care Lactate Increases the Prognostic Accuracy of National Early Warning Score 2 for Early Risk Stratification of Mortality: Results of a Multicenter, Observational Study

**DOI:** 10.3390/jcm9041156

**Published:** 2020-04-18

**Authors:** Francisco Martín-Rodríguez, Raúl López-Izquierdo, Juan F. Delgado Benito, Ancor Sanz-García, Carlos del Pozo Vegas, Miguel Ángel Castro Villamor, José Luis Martín-Conty, Guillermo J. Ortega

**Affiliations:** 1 Advanced Clinical Simulation Center, School of Medicine, Universidad de Valladolid, 47005 Valladolid, Spain; fmartin@saludcastillayleon.es (F.M.-R.); mcastrovi@saludcastillayleon.es (M.Á.C.V.); 2 Advanced Life Support Unit, Emergency Medical Services, 47007 Valladolid, Spain; jdelgado@saludcastillayleon.es; 3 Emergency Department, Hospital Universitario Rio Hortega, 47012 Valladolid, Spain; 4 Data Analysis Unit, Health Research Institute, Hospital de la Princesa, 28006 Madrid, Spain; ancor.sanz@gmail.com (A.S.-G.); agetro.ortega@gmail.com (G.J.O.); 5 Emergency Department, Hospital Clínico Universitario de Valladolid, 47005 Valladolid, Spain; cpozove@saludcastillayleon.es; 6 Faculty of Health Sciences, Universidad de Castilla la Mancha, 45600 Talavera de la Reina, Ciudad Real, Spain; JoseLuis.MartinConty@uclm.es; 7 CONICET, C1425FQB Buenos Aires, Argentina

**Keywords:** biomarkers, clinical decision-making, clinical deterioration, early warning, emergency medical services, mortality, patient safety

## Abstract

The objective of this study was to assess whether the use of prehospital lactate (pLA) can increase the prognostic accuracy of the National Early Warning Score 2 (NEWS2) to detect the risk of death within 48 h. A prospective, multicenter study in adults treated consecutively by the emergency medical services (EMS) included six advanced life support (ALS) services and five hospitals. Patients were assigned to one of four groups according to their risk of mortality (low, low-medium, medium, and high), as determined by the NEWS2 score. For each group, the validity of pLA in our cohort was assessed by the area under the curve (AUC) of the receiver operating characteristic (ROC) curve. In this study, 3081 participants with a median age of 69 years (Interquartile range (IQR): 54–81) were included. The two-day mortality was 4.4% (137 cases). The scale derived from the implementation of the pLA improved the capacity of the NEWS2 to discriminate low risk of mortality, with an AUC of 0.910 (95% CI: 0.87–0.94; *p* < 0.001). The risk stratification provided by the NEWS2 can be improved by incorporating pLA measurement to more accurately predict the risk of mortality in patients with low risk.

## 1. Introduction

Assistance to people with acute disease in the prehospital context has evolved rapidly in recent years, making it possible to diagnose and treat bedside pathologies on many occasions [1]. Thanks to research in this field and to the implementation of specific protocols and technological developments, we now have tools that can be employed at the scene or on the road, which can help to evaluate the actual state of a patient from the very first contact of the emergency medical services (EMS) until the arrival at the emergency department (ED) [2].

The use of early warning scores (EWS) is a reality in the current clinical practice [3] and has become a standard procedure in many contexts and pathologies [4,5]. Among all scores, the National Early Warning Score 2 (NEWS2) [6] has proven to be useful in the prehospital context, is validated, and commonly used internationally [7,8,9]. Moreover, technological development has led to the use of rapid-response point-of-care testing [10,11], as such is the case of prehospital lactate (pLA), which is a sensitive indicator of tissue hypoperfusion, reliable, and easy to obtain [12,13].

One challenge faced by the EMS is the capability of detecting risk of mortality in patients with acute diseases [14], fundamentally in time-dependent pathologies where a diagnostic or therapeutic delay may negatively influence the outcome, thereby increasing morbidity and mortality [15]. In addition, these pathologies may not be suspected or detected initially. In these contexts, the use of any aid that can discriminate the risk of mortality should be evaluated because it can reduce the interval for definitive qualified health care [16].

The use of EWS and biomarkers represents a fundamental tool that enables the systematized evaluation of the patient and can predict possible serious adverse events (SAEs), individualizing the assessment and care of each patient [17]. EWS and biomarkers have been used separately and in combination [18,19]. Specifically, the relationship between the NEWS2 and lactate has been analyzed in diverse clinical contexts [20,21,22].

Our main objective was to analyze whether the use of pLA increases the prognostic capacity of the NEWS2 to predict the risk of early mortality in people with acute disease treated by EMS.

## 2. Material and Methods

### 2.1. Study Design

We conducted a prospective, multicenter study in adults treated consecutively by the EMS and transported in advanced life support units (ALS) to referral hospitals between 1 October 2018 and 3 November 2019.

The present study was in accordance with Good Clinical Practice and the Declaration of Helsinki. The Research Ethics Committee of each participating institution approved the protocol. (REC number: #PI 18-010, #PI 18-895, #PI 18-10/119, #PI MBCA/dgc and #PI 2049). All patients (or guardians) signed an informed consent. The study was coordinated by the Advanced Clinical Simulation Center of the Faculty of Medicine at Valladolid University. This study is reported in line with the STROBE statement.

### 2.2. Study Setting

The study was conducted in six ALS and five hospitals (four tertiary university hospitals and one small general district hospital) of the public health system of Castile and Leon (Spain) with a reference population of 1,364,952 inhabitants.

The sample size calculation was performed using an area under the curve (AUC) of the expected receiver operating characteristic (ROC) of 90% with a confidence level of 99% and an accuracy of 1%, with 2543 subjects. We estimated a follow-up loss in 5%, so the estimated final sample size was 2677 subjects, for an early mortality rate of 4%, in line with similar studies [7,11,18].

Each ALS was composed of a medical doctor, an emergency registered nurse (ERN), and two paramedics. These units operate nonstop 24 h a day, 365 days a year, performing standard life support maneuvers on the scene or on the road.

### 2.3. Population

Eligible patients were recruited from among all phone requests for assistance during the study period. Inclusion criteria were age over 18 years and being transported in ALS to the ED of the reference hospital after the initial evaluation and life support maneuvers on the scene.

We excluded patients in cardiorespiratory arrest or with lesions incompatible with life, pregnant women, people evacuated by other means of transport (e.g., basic life support) or discharged in situ. Also, we excluded from the present study the following cases: Impossibility of obtaining informed consent at the scene or at the hospital; lack of exactness in the patient data; cases with evident physical risk at the scene, like people with psychiatric problems, attacks, armed incidents, and accidents of any etiology with ongoing risks.

### 2.4. Study Protocol

The review protocol of this study was registered with ICTRP (doi.org/10.1186/ISRCTN17676798).

The main outcome variable was early hospital mortality (within 48 h) from any cause—globally and by stratified groups—according to the NEWS2 estimated risk band. Patients discharged prior to 48 h (dead or alive in this interim) and those who died before the arrival of the EMS were not included in the study.

The EMS collects all clinical data necessary to perform the NEWS2 as a standard procedure. Based on the history, exploration, and complementary tests and, depending on the type of incident, they decide the best strategy for each case accordingly with the current management guidelines for the pathology in emergencies. In addition, for this study, the determination of pLA was introduced in all ALS participating in the study, but since it was an observational study, the value of pLA was not taken into account for clinical decision-making. All the variables considered in the present work were recorded at the event site or during transportation by the EMS.

The NEWS2 calculation was performed according to the recommendations of The Royal College of Physicians of London [6]. The NEWS2 analyzes seven clinical parameters: Respiratory rate, oxygen saturation, use of supplemental oxygen, heart rate, systolic blood pressure, temperature, and level of consciousness (Table 1). Partial scores for each category are added to obtain an overall score. Depending on this score’s value, the risk of mortality was stratified in the following way: 1–4 points as ‘low risk’, score of 3 in any individual parameter as ‘low-medium risk’, aggregate score 5–6 as ‘medium risk’, and aggregate score of 7 or more as ‘high risk’ [6].

Respiratory rate was determined by direct observation by counting respiratory cycles in one minute. Oxygen saturation, systolic blood pressure, and heart rate were measured with the LifePAK^®^ 15 defibrillator monitor (Physio-Control, Inc., Redmond, WA, USA). Temperature was determined with the ThermoScan^®^ PRO 6000 tympanic thermometer (Welch Allyn, Inc, Skaneateles Falls, NY, USA). EMS also recorded both the use of supplemental oxygen at their arrival to the event site and the patient mental state (Glasgow Coma Scale (GCS) of less than 15 points or new confusion *en route*). GCS was converted to the AVPU scale by considering GCS < 15 as V, P, or U categories of the AVPU [23], as shown in Table 1.

Blood, and therefore lactate, were obtained from all the patients in the study through a venous line, which was necessary in all cases. An Accutrend Plus measuring device (Roche Diagnostics, Mannheim, Germany) with a measuring range of 0.8–21.7 mmol/L was used to determine pLA values. The protocol for obtaining the pLA involves five phases. First, the Accutrend Plus device is turned on, the test strip is inserted, and the verification code on the screen is checked against the code of the reagent canister, as well as the expiration date. Second, blood is taken from the vein with a 1-mL syringe. Third, between 15 μL and 40 μL of blood is deposited on the test strip inside the Accutrend Plus. Fourth, the lid is closed, and the pLA value in mmol/L appears on the screen within 60 s. Then, the lid is opened, the reagent removed, and the device cleaned. The time taken from the blood sampling to its placement in the device should not exceed 1 min.

To guarantee the traceability of all analyses, we recorded all reagents used in the study: Lot number, expiration date, and verification code. All Accutrend Plus devices used in the study were calibrated by the researchers from each participating center every 25 determinations using the Accutrend^®^ BM-Control-Lactate control solution (Roche Diagnostics, Mannheim, Germany).

### 2.5. Data Abstraction

Prior to the field phase, all members of the research team received specific training about the standardized way of obtaining the necessary data for the calculation of the NEWS2 [6]: Use, maintenance, and calibration of electromedical equipment; blood extraction protocol; and pLA determination.

All data were recorded in a standardized case form (standard clinical history used by the EMS). During the first EMS contact with the patient, the ERN recorded: Administrative data; times of arrival, assistance, and evacuation; reason for calling according to the Advanced Medical Priority Dispatch System [24,25]; the set of vital signs (respiratory rate, oxygen saturation, use of supplemental oxygen, heart rate, systolic blood pressure, temperature, and level of consciousness); and the pLA value.

With the whole set of vital signs, the total NEWS2 score was obtained and the risk of mortality was finally stratified [6].

Forty-eight hours after the index event, an associated researcher from each hospital reviewed the electronic medical record through the database of the public health system (JIMENA-SACYL) and obtained the hospital outcomes: need for admission and/or intensive care unit (ICU) and early hospital mortality (within 48 h). Only mortality was used for the analysis, and the other outcomes were used only for descriptive purposes. To guarantee an exact link of the data between the EMS history and the electronic history in each case, the necessary linking criteria were date, time of arrival, ALS code, name and surname, sex, and age.

### 2.6. Data Analysis

All patient data were recorded electronically in a database created specifically for this purpose. The case registration form was tested to eliminate ambiguous elements and to validate the data collection instrument.

Categorical variables were expressed as number of patients and percentage (%), continuous variables were expressed as median and interquartile range (IQR) after normal distribution was discarded by a Shapiro–Wilk test. Among all registered parameters, the variables used in the analyses were, as the outcome variable: mortality within two days, and as the predictor variables: NEWS2 and pLA. None of the variables presented missing data. The statistical comparison of the cohort characteristics between groups and for each variable was performed using Student’s *t*-test or Chi-square test when required [26].

**Figure 1:**
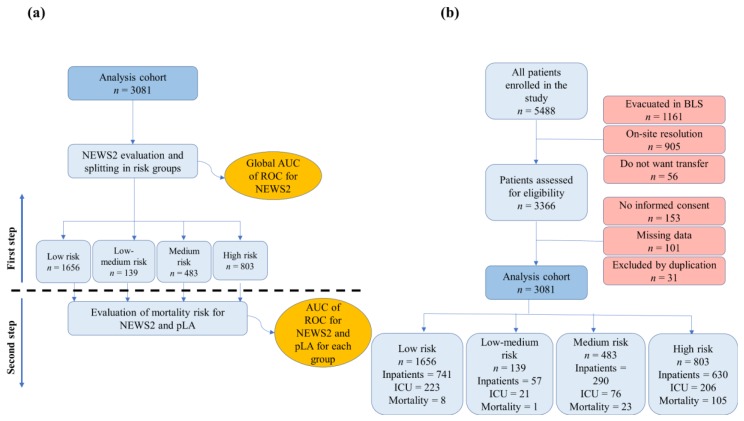
(**a**) The two-step analysis procedure. (**b**) Epidemiology diagram showing analysis population by groups derived from the risk stratification provided by the NEWS2. AUC: Area under the curve; BLS: Basic life support; NEWS2: National Early Warning Score 2; pLA: Prehospital lactate; ROC: Receiver operating characteristic.

A two-step sequential procedure (Figure 1a) was followed for the analysis of the data. In the first step, an assessment of the obtained NEWS2 classification against the outcome (mortality risk) through the AUC of the ROC for all the patients was performed and, following the recommendations of the Royal College of Physicians of London, the whole cohort was divided in four groups according to the obtained NEWS2 score. This first evaluation allows not only checking the NEWS2 stratification, but also splitting the cohort in four risk groups. In the second step of the procedure, on each of the obtained groups, a new evaluation of mortality risk was performed using both the pLA levels, and, independently, the NEWS2 again. This second-step NEWS2 and pLA scores applied on each of the previously obtained groups were not combined into a single score, but they were instead considered separately in order to compare their performance regarding mortality. In this way, both systems were applied to each risk group (low, low-medium, medium, and high) to determine their capability of predicting mortality, and, subsequently, their corresponding results were compared.

The validity of NEWS2 and pLA in the four groups was assessed by the AUC of the ROC curve for predicting mortality within two days. In particular, the NEWS2 value or pLA levels were used in a logistic regression model to predict the probability of mortality given the value or levels of individual patients. Then, to evaluate the discrimination between outcomes (deceased or alive,) the AUC of the ROC curve was used. In addition to the AUC, a 95% confidence interval (95% CI) and a *p*-value corresponding to the comparison against the chance level (AUC = 0.5) were calculated. The adjusted AUC was estimated as an internal validation procedure computed with 300 stratified bootstrap replicates, which is the recommended method for a predictive logistic regression model validation [27]. The database use for the calculations can be found in the supplementary data. Further details of the predictive model were also estimated including the odds ratios of pLA and NEWS2. A statistical comparison between the corresponding AUCs of both variables for each group was finally obtained.

All statistical analyses were performed using our own codes and base functions in R, version 3.5.1 (http://www.R-project.org; the R Foundation for Statistical Computing, Vienna, Austria).

## 3. Results

### 3.1. Patients

In the study period, 5488 cases were examined for eligibility and 3081 participants met the inclusion criteria (Figure 1).

Median age was 69 years (Interquartile range (IQR) 54–81 years), and 1269 (41.2%) were women. Demographic characteristics and their statistical differences are described in Table 2. The two-day mortality was 4.4% (137 cases), most commonly of cardiovascular origin (46 cases, 33.5%). The differences between survivors and nonsurvivors were significant in all the parameters that make up the NEWS2, such as it is the case of pLA or the need for ICU (71 cases, 51.8%) (Table 2).

Medical pathologies (2736 cases, 88.8%) far exceeded trauma cases (345 cases, 11.2%). Table 3 shows the reasons for requesting assistance.

### 3.2. Patient Classification and Global NEWS2 Performance

**Figure 2:**
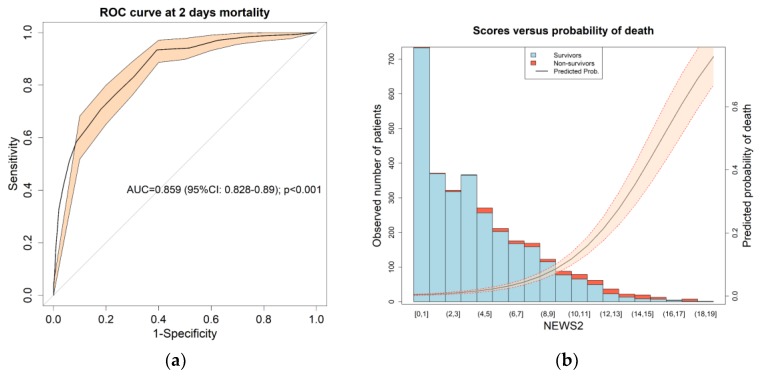
(**a**) Receiver operational characteristic (ROC) by NEWS2 for early mortality. The bold line shows the value of the ROC curve and the orange shading is the 95% confidence interval. In the center of the graph is the area under the curve (AUC), its 95% confidence interval and the *p*-value of the comparison against the null hypothesis (AUC = 0.5). (**b**) Probability of death based on the value of NEWS2 for early mortality. The bar graph shows the number of patients in the whole cohort for each scale value (survivors in blue and nonsurvivors in red). The trend line shows the estimated probability of death.

Before splitting the sample in the risk groups, the NEWS2 performance to predict mortality was evaluated presenting an AUC of 0.859 (95% CI: 0.82–0.89; *p* < 0.001; Figure 2a). The internally validated AUC was 0.859 (95% CI: 0.85–0.86). As can be seen in Figure 2b, the highest NEWS2 values correspond to an increase in mortality within two days, also shown by the predicted probability trend line.

Following the recommendations of The Royal College of Physicians of London, patients were divided into four groups according to the risk of mortality (Table 4): Low (1656 cases, 8 nonsurvivors), low-medium (139 cases, 1 nonsurvivor), medium (483 cases, 23 nonsurvivors), and high (803 cases, 105 nonsurvivors).

### 3.3. Comparison of Mortality Discrimination Derived from pLA and NEWS2

**Figure 3:**
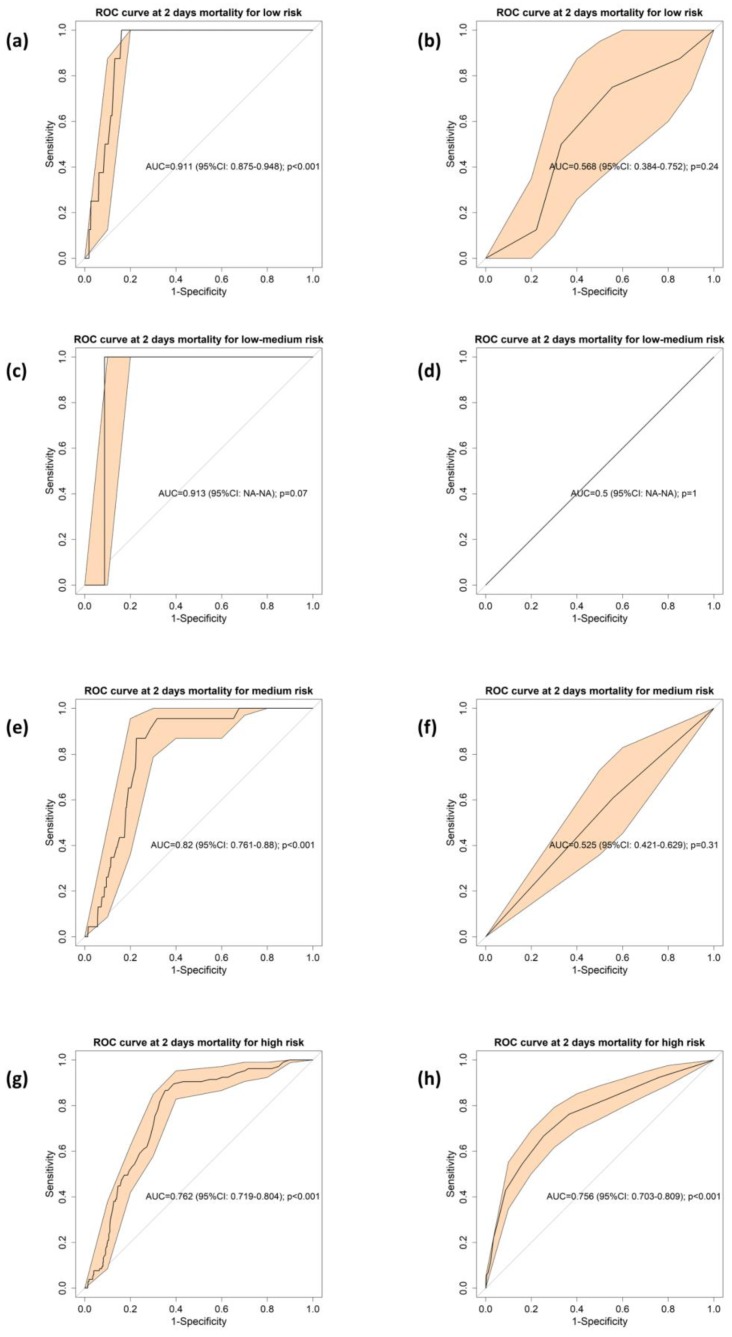
Receiver operational characteristic (ROC) of pLA for early mortality based on the stratification of the risk of mortality. (**a**) Low risk, (**c**) low-intermediate risk, (**e**) intermediate risk, (**g**) high risk; and ROC of NEWS2 for early mortality based on the stratification of the risk of mortality (**b**) low risk, (**d**) low-intermediate risk, (**f**) intermediate risk, (**h**) high risk. The bold line shows the value of the ROC curve and the orange shading is the 95% confidence interval. In the center of the graph is the area under the curve (AUC), its 95% confidence interval, and the *p*-value of the comparison against the null hypothesis (AUC = 0.5).

The AUCs of the pLA and the NEWS2 for each risk group obtained in the step one of the procedure—initial NEWS2 stratification—are shown in Figure 3. For the groups of low, low-medium and medium mortality risk, the scale derived from the implementation of the pLA improved the results of NEWS2 discrimination, as evidenced by the results of pLA: AUC 0.911 (95% CI: 0.87–0.95; *p* < 0.001; Figure 3a), 0.913 (95% CI: NA–NA; *p* = 0.07; Figure 3c), and AUC 0.820 (95% CI: 0.76–0.88; *p* < 0.001; Figure 3e); and for NEWS2: AUC 0.568 (95% CI: 0.38–0.75; *p* = 0.24; Figure 3b), 0.5 (95% CI: NA–NA; *p* = 1; Figure 3d), and AUC 0.525 (95% CI: 0.42–0.62; *p* = 0.31; Figure 3f).

In contrast, for the case of the high-risk group, the implementation of the pLA was nearly similar to NEWS2, that is, 0.762 (95% CI: 0.71–0.80; *p* < 0.001; Figure 3g), and 0.756 (95% CI: 0.70–0.80; Figure 3h), respectively. The comparison of AUCs between pLA and NEWS2 showed statistically significant differences between AUCs of low- (*p* < 0.001), low-medium- (*p* < 0.001), and medium-risk (*p* < 0.001) groups but not for the high-risk group (*p* = 0.86).

In all cases, the odds ratios of pLA were statistically significant, with values of 1.29 for low-risk and 1.15 for medium- and high-risk groups (Table 5), except for low-medium, but for NEWS2, only high-risk presented a significant value of 1.42 (Table 5). Both tables present further measures of both scores.

Additionally, in order to exclude those patients with altered mental status, we evaluated the performance of NEWS2 and pLA in patients with NEWS2 < 3. Again, the pLA outperformed NEWS2, AUC 0.908 (95% CI: 0.856–0.96; *p* < 0.001) and 0.555 (95% CI: 0.215–0.896; *p* = 0.34), respectively. The comparison of AUCs showed statistically significant differences (*p* < 0.02). Similarly, if we consider the cutoff value for patients with NEWS2 < 3 as a threshold to determine the mortality, our results showed that all deaths occurred in patients with pLA > 4 mmol/L. Likewise, this value was similar to the one found in low-risk group (Table 5). In this sense, further investigation on the relationship of that pLA nonmortality can be seen in Table 6. This table shows the percentage of mortality in each group that resulted from the combination of NEWS2 above or below 3 and pLA above or below 4 mmol/L, and also highlights the low percentage of mortality for those patients with NEWS2 > 3 but also with less than 4 mmol/L.

## 4. Discussion

In this prospective, multicenter study, we evaluated the ability of pLA to increase the prognostic accuracy of the NEWS2 for determining early mortality. We found that after performing a stratification of the risk of mortality provided by the NEWS2, a subsequent pLA measurement helps to discriminate more efficiently the risk of patients categorized as low risk.

It is common to find studies that analyze the use of EWS and lactate in people with acute disease [18,19,28] and studies that specifically analyze the association between the NEWS2 and lactate [20,21,29,30]. However, it is rather uncommon to find studies focused on the prehospital context [31,32,33], especially dealing with the use of the pLA for a further evaluation of risk groups stratified by the NEWS2.

The regular assessment of the risk of mortality in patients with acute disease and the stratification of the risk offered by the NEWS2 provides the EMS with a more precise knowledge of the situation [34,35], it can helps in setting treatment goals, and even it can guide them toward the most appropriate destination center. Therefore, risk stratification can help in decision-making at critical moments.

The current recommendations of the Royal College of Physicians of London [6] set thresholds and triggers. An aggregate score of 7 or more indicates high risk that demands the response of the critical care team [36]. With this strict criterion for activating the emergency response, health systems ensure that no ill person is at risk of mortality and of suffering SAEs. The NEWS2 has a high sensitivity in patients with medium and high risk, and its clinical utility is certainly undebatable. Our study shows that by incorporating a subsequent assessment of the pLA, a better characterization of the risk of mortality in patients with low risk is achieved. In this way, care professionals may know in advance the severity of the condition they are confronting and the actual need for evacuation, which certainly may facilitate the decision-making process [9,37].

The use of point-of-care testing, and specifically pLA, is becoming widespread among the EMS [11,38] because it provides a robust clinical and prognostic utility with high reliability [39]. The technical possibility of performing bedside analytics allows the incorporation of these devices into standard procedures of EMS, clearly helping in decision-making.

Our data indicate that a pLA less than 4 mmol/L in people with acute disease, categorized with low risk, but also for those with NEWS2 < 3, indicates a minimal risk of mortality. In contrast, a pLA greater than 4 mmol/L, despite a low risk, is associated with a significant increase in mortality, which it was also seen for patients with NEWS2 > 3, a fact that should be taken into account by professionals when predicting early SAEs, even in those *a priori* less serious situations [40,41].

The NEWS2 is easy to use, validated, and widely implemented. Likewise, measuring pLA has become easier and cheaper in the last years. For both parameters, their clinical utility as a bedside tool is undebatable. The portability of the NEWS2 and pLA makes their isolated or combined use an excellent tool for EMS either at the scene or on the road.

In the prehospital context, professionals must make rapid and critical decisions, even with little data at hand. After a brief history and rapid examination and guided by few diagnostic tests, the objective must be decided according to the management guidelines of each pathology. Both the objective and a structured clinical evaluation are the main tasks the EMS should perform. The uses of EWS and point-of-care testing procedures could be included in their routine. In this case, scores certainly may be of help.

### Strength and Limitations

The strength of the present study rests mainly in the methodology itself, as it is a multicenter prospective study with an extensive though very homogeneous cohort of participants (based on restrictive inclusion criteria). Although all personnel are professionals with extensive experience in the prehospital activity and received prior training, the work presented here possesses some limitations. On the one hand, patients’ selection bias exists because they were selected based on the opportunity criteria in a specific period of time. Also, by including only patients evaluated and evacuated in ALS, a high number of older adults were involved. This fact nonetheless reflects the target population of our geographic area, and it is in line with similar studies [9]. On the other hand, because data extraction was done on a nonblinded procedure, the cases were only considered if the mortality occurred—due to any cause—within the hospital and within the first 48 h after EMS care, in order to minimize the selection bias. The principal investigator made monthly visits to all ALS and all ED to verify the correct implementation of the research protocol with the objective to resolve potential discrepancies. The principal investigator reviewed 50% of the sample data and all cases of early mortality.

## 5. Conclusions

The risk stratification provided by the NEWS2 can be improved by incorporating pLA determination to more accurately predict the risk of mortality in patients with low risk.

## Figures and Tables

**Table 1:** National Early Warning Score 2 (NEWS2).

NEWS2	3	2	1	0	1	2	3
Pulse (bpm)	≤40		41–50	51–90	91–110	111–130	≥131
BR (bpm)	≤8		9–11	12–20		21–24	≥25
T (°C)	≤35		35.1–36	36.1–38	38.1–39	≥39.1	
SBP (mmHg)	≤90	91–100	101–110	111–219			≥220
SpO_2_ (%) Scale 1	≤91	92–93	94–95	≥96			
SpO_2_ (%) Scale 2 ^1^	≤83	84–85	86–87	88–92 ≥93 air	93–94 Oxygen	95–96 Oxygen	≥97 Oxygen
Air oxygen		Oxygen		Air			
AVPU (scale)				A			V, P, U

^1^ In patients with hypercapnic respiratory insufficiency, scale 2 should be used to weight the oxygen saturation score. SBP: Systolic blood pressure; BR: Breathing rate; SpO_2_: Oxygen saturation; AVPU: Alert, verbal, pain, unresponsive: T: Temperature. Reproduced from The Royal College of Physicians. National Early Warning Score (NEWS) 2: Standardizing the assessment of acute-illness severity in the NHS. Updated report of a working party. London: RCP, 2017 [6].

**Table 2:** Demographic, prehospital, and hospital characteristics of study subjects (death statistics refer to mortality rates in less than 48 h).

Characteristic ^1^	Total	Survivors	Non-Survivors	*p*-Value
Number (*n* (%))	3081 (100)	2944 (95.6)	137 (4.4)	
Age (years)	69 (54–81)	69 (53–81)	80 (65–87)	0.001 ^2^
Female	1269 (41.2)	1218 (41.4)	51 (37.2)	0.335 ^3^
Isochronous (Minutes)				
Arrival time	10 (8–13)	10 (8–13)	10 (8–15)	0.184 ^2^
Support time	28 (22–34)	28 (22–34)	32 (25–39)	0.001 ^2^
Transfer time	10 (7–14)	10 (7–13)	10 (7–18)	0.039 ^2^
Initial Evaluation				
NEWS 2 (points)	4 (2–7)	4 (2–6)	11 (7–13)	0.001 ^2^
Breathing rate (bpm)	17 (14–24)	17 (14–22)	23 (14–34)	0.001 ^2^
Supplemental oxygen	389 (12.6)	343 (11.7)	46 (33.6)	0.001 ^2^
Oxygen saturation (%)	96 (93–98)	96 (93–98)	83 (72–93)	0.001 ^2^
Heart rate (bpm)	85 (70–104)	84 (70–102)	97 (71–121)	0.009 ^2^
SBP (mmHg)	137 (118–155)	137 (119–155)	124 (93–153)	0.002 ^2^
Temperature (°C)	36.3 (36.0–36.8)	36.3 (36.0–36.8)	36.0 (35.1–37.0)	0.014 ^2^
GCS (3–15 points)	15 (15–15)	15 (15–15)	11 (3–15)	0.001 ^2^
pLA (mmol/L)	2.9 (2.0–4.0)	2.8 (1.9–3.8)	5.1 (4.3–7.3)	0.001 ^2^
Hospital Outcomes				
Inpatients	1718 (55.8)	1581 (53.7)	137 (100)	0.001 ^2^
ICU	526 (17.1)	455 (15.5)	71 (51.8)	0.001 ^2^

^1^ Values expressed as total number (fraction) and medians (25th percentile–75th percentile) as appropriate. ^2^ The *p*-values were calculated with Mann–Whitney U-test. ^3^ The *p*-values were calculated with Chi-square test. NEWS 2: National Early Warning Score 2; SBP: Systolic blood pressure; GCS: Glasgow coma scale; pLA: Point-of-care lactate; ICU: Intensive care unit.

**Table 3:** Distribution of cases according to the Advanced Medical Priority Dispatch System (death statistics refer to mortality rates in less than 48 h).

Pathologies	Total	Survivors	Non-Survivors
Abdominal Pain/Problems	176 (5.7)	166 (5.6)	10 (7.3)
Allergies (Reactions)	33 (1.1)	33 (1.1)	0
Animal Bites/Attacks	3 (0.1)	3 (0.1)	0
Assault/Sexual Assault/Stun Gun	2 (0.1)	1 (0)	1 (0.7)
Breathing Problems	333 (10.8)	311 (10.6)	22 (16.1)
Burns (Scalds)/Explosions	7 (0.2)	6 (0.2)	1 (0.7)
Carbon Monoxide/Inhalation	12 (0.4)	12 (0.4)	0
Chest Pain	593 (19.2)	579 (19.7)	14 (10.2)
Choking	17 (0.6)	12 (0.4)	5 (3.6)
Convulsions/Seizures	185 (6.0)	185 (6.3)	0
Diabetic Problems	37 (1.2)	36 (1.2)	1 (0.7)
Electrocution/Lightning	2 (0.1)	2 (0.1)	0
Falls	59 (1.9)	57 (1.9)	2 (1.5)
Headache	64 (2.1)	64 (2.2)	0
Heart Problems/AICD	381 (12.4)	349 (11.9)	32 (23.4)
Heat/Cold Exposure	11 (0.4)	10 (0.3)	1 (0.7)
Hemorrhage/Lacerations	27 (0.9)	27 (0.9)	0
Inaccessible Incident/Entrapments	3 (0.1)	3 (0.1)	0
Overdose/Poisoning (Ingestion)	158 (5.1)	154 (5.2)	4 (2.9)
Sick Person	106 (3.4)	96 (3.3)	10 (7.3)
Stab/Gunshot/Penetrating Trauma	10 (0.3)	10 (0.3)	0
Stroke/Transient Ischemic Attack	301 (9.8)	290 (9.9)	11 (8.0)
Traffic/Transportation Incidents	160 (5.2)	155 (5.3)	5 (3.6)
Traumatic Injuries	49 (1.6)	44 (1.5)	5 (3.6)
Unconscious/Fainting (Near)	352 (11.4)	339 (11.5)	13 (9.5)

Values expressed as total number and percentage of mortality in parentheses; AICD: Automated Implantable Cardioverter-Defibrillator.

**Table 4:** Characteristics of study subjects by risk groups based on the NEWS2.

Variable	Mortality Risk			
	Low	Low-Medium	Medium	High
Number	1656 (53.8)	139 (4.5)	483 (15.7)	803 (26.1)
Age (years)	65 (51–78)	66 (49–79)	72 (55–83)	76 (62–84)
Female	669 (52.7)	67 (5.2)	208 (16.4)	325 (25.6)
pLA (mmol/L)	2.4 (1.8–3.4)	2.9 (2.0–3.8)	3.1 (2.2–4.4)	3.7 (2.6–5.1)
Hospital Outcomes				
Inpatients	741 (44.7)	57 (41)	290 (60.0)	630 (78.5)
ICU	223 (13.4)	21 (15.1)	76 (15.7)	206 (25.7)
Mortality	8 (0.5)	1 (0.7)	23 (4.8)	105 (13.1)

For Number of patients, sex, and hospital outcome values expressed as total number (fraction) and for the other values expressed medians (25th percentile–75th percentile). pLA; Prehospital lactate; ICU: Patients admitted to intensive care unit; Inpatients: Patients admitted to hospital (including those admitted to ICU); Mortality: Patients who died within 48 h. Low risk: Aggregate score 0–4. Low-medium risk: Score of 3 in any individual parameter. Medium risk: Aggregate score 5–6. High risk: Aggregate score 7 or more.

**Table 5:** Measures of the predictive model.

	**Mortality Risk by pLA**			
	**Low**	**Low-Medium**	**Medium**	**High**
Prev.	0.005	0.005	0.048	0.131
pLA cut-off	4.0	4.9	4.3	4.1
Se	28.3 (8.83–47.7)	50 (12.2–87.7)	30.4 (11.7–49.1)	28.4 (11.5–45.4)
Sp	86.4 (73.1–99.6)	71.2 (45.1–97.3)	81.5 (67.3–95.6)	81.0 (67.4–94.6)
PPV	1.01 (0.37–1.65)	1.35 (0–3.08)	7.46 (5.25–9.67)	15.2 (10.6–19.7)
NPV	99.6 (99.5–99.7)	99.5 (99.2–99.8)	96.2 (95.4–97.1)	89.4 (87.4–91.3)
LR+	2.14 (0.78–3.51)	1.97 (0–4.55)	1.66 (1.15–2.17)	1.29 (0.87–1.7)
LR−	0.77 (0.58–0.95)	0.57 (0.15–1)	0.77 (0.59–0.95	0.80 (0.63–0.95
DA	86.1 (73–99.2)	71.1 (45.3–96.8)	79.1 (66.4–91.7)	74,1 (64.3–83.9)
OR	1.27 (1.12–1.45) (*p* < 0.001)	NS	1.15 (1.06–1.24) (*p* < 0.001)	1.15 (1.10–120) (*p* < 0.001)
	**Mortality Risk by NEWS 2**			
	**Low**	**Low-Medium**	**Medium**	**High**
Prev.	0.005	NA	0.048	0.131
NEWS2 cut-off	2	NA	5	11
Se	65 (21.9–100)	NA	69.5 (0–100)	66.7 (57.2–75)
Sp	40.8 (0–81.9)	NA	27 (0–100)	74.6 (71.3–77.7)
PPV	0.52 (0.30–0.74)	NA	4.5 (1.22–7.78)	28.3 (23.1–34.3)
NPV	99.6 (99.4–99.8)	NA	94.8 (NA–NA)	93.7 (91.4–95.4)
LR+	1.09 (0.63–1.54)	NA	0.94 (0.22–1.66)	2.63 (2.18–3.17)
LR−	0.81 (0.44–1.19)	NA	1.08 (NA–NA)	0.45 (0.34–0.59)
DA	40.9 (18.9–81.6)	NA	26.9 (0–100)	73.6 (70.4–76.5)
OR	NS	NS	NS	5.89 (3.79–9.14) (*p* < 0.001)

Bracketed numbers indicate 95% confidence interval. Prev: Prevalence; pLA: Prehospital lactate; Se: Sensitivity; Sp: Specificity; PPV: Positive predictive value: NPV: Negative predictive value; LR: Likelihood ratio; OR: Odds ratio; DA: Diagnostic accuracy. Low risk: Aggregate score 0–4. Low-medium risk: Score of 3 in any individual parameter. Medium risk: Aggregate score 5–6. High risk: Aggregate score 7 or more.

**Table 6:** Mortality of groups resulted from the combination of NEWS2 above and below 3 and prehospital lactate above and below the cutoff (4 mmol/L).

		Survivors	Non-Survivors	Odd Ratios
NEWS2 < 3	pLA < 4	944 (100%)	0 (0%)	*
	pLA > 4	158 (97.5%)	4 (2.5%)	
NEWS2 > 3	pLA < 4	1075 (98.5%)	17 (1.5%)	15.75(9.27–28.32) *p* < 0.001
	pLA > 4	449 (80%)	112 (20%)	

Bracketed numbers indicate 95% confidence interval. pLA: Prehospital lactate; * cannot be calculated because of 0 cases in one group.

## Supplementary Materials

The following are available online at https://www.mdpi.com/2077-0383/9/4/1156/s1, Table S1: Database used in the present work.
